# A Large Number of Nuclear Genes in the Human Parasite *Blastocystis* Require mRNA Polyadenylation to Create Functional Termination Codons

**DOI:** 10.1093/gbe/evu146

**Published:** 2014-07-10

**Authors:** Vladimír Klimeš, Eleni Gentekaki, Andrew J. Roger, Marek Eliáš

**Affiliations:** ^1^Department of Biology and Ecology, Faculty of Science, University of Ostrava, Czech Republic; ^2^Department of Biochemistry and Molecular Biology, Dalhousie University, Halifax, Nova Scotia, Canada; ^3^Integrated Microbial Biodiversity Program, Canadian Institute for Advanced Research, Halifax, Nova Scotia, Canada

**Keywords:** *Blastocystis*, evolution, gene expression, mRNA processing, polyadenylation, termination codons, translation

## Abstract

Termination codons in mRNA molecules are typically specified directly by the sequence of the corresponding gene. However, in mitochondria of a few eukaryotic groups, some mRNAs contain the termination codon UAA deriving one or both adenosines from transcript polyadenylation. Here, we show that a similar phenomenon occurs for a substantial number of nuclear genes in *Blastocystis* spp., divergent unicellular eukaryote gut parasites. Our analyses of published genomic data from *Blastocystis* sp. subtype 7 revealed that polyadenylation-mediated creation of termination codons occurs in approximately 15% of all nuclear genes. As this phenomenon has not been noticed before, the procedure previously employed to annotate the *Blastocystis* nuclear genome sequence failed to correctly define the structure of the 3′-ends of hundreds of genes. From sequence data we have obtained from the distantly related *Blastocystis* sp. subtype 1 strain, we show that this phenomenon is widespread within the *Blastocystis* genus. Polyadenylation in *Blastocystis* appears to be directed by a conserved GU-rich element located four nucleotides downstream of the polyadenylation site. Thus, the highly precise positioning of the polyadenylation in *Blastocystis* has allowed reduction of the 3′-untranslated regions to the point that, in many genes, only one or two nucleotides of the termination codon are left.

## Introduction

The coding region of a mature mRNA molecule corresponds directly to the sequence of a primary transcript of a gene once intronic sequences are removed. However, some gene expression systems are more complex, and are most frequently reported within organelles of various eukaryotes. Unorthodox mechanisms for producing a functional coding sequence include posttranscriptional editing, found in mitochondrial, nuclear and plastid genes (Knoop 2011; Dorrell and Howe 2012; Takenaka et al. 2013), and *trans*-splicing recently discovered in some protist mitochondria (Moreira et al. 2012; Jackson and Waller 2013; Kiethega et al. 2013) and the nucleus of the diplomonad *Giardia intestinalis* (Kamikawa et al. 2011).

An interesting form of posttranscriptional sequence modification was described in mammalian mitochondria, in which part of the termination codon UAA of some mRNAs is created by polyadenylation (Anderson et al. 1981; Chang and Tong 2012); such mature transcripts therefore lack a 3′-untranslated region (3′-UTR). A similar phenomenon was also described for mRNAs of the *cox3* gene in the mitochondria of dinoflagellates (Jackson et al. 2007) and at least for some mRNAs encoded by the mitochondrial genome of the euglenozoan *Diplonema papillatum* (Kiethega et al. 2013). On the other hand, the occurrence of this phenomenon in nuclear transcripts seems to be very rare. To our knowledge, the only reported cases concern a few mammalian genes that employ alternative polyadenylation sites. One such case is the mouse thymidylate synthase gene that has multiple mRNA isoforms. The less abundant isoform exhibits the conventional gene-encoded termination codon UAG followed by a 3′-UTR and the poly(A) tail, whereas the more abundant isoform uses an alternative polyadenylation site that changes the UAG codon to UAA followed directly by the poly(A) tail (Jenh et al. 1986). Another case involves a mechanism dubbed polyadenylation-mediated Tyr-to-stop codon conversion (PAY*) that is used for several genes including glutamyl-prolyl tRNA synthetase. In this mechanism, an mRNA molecule is alternatively cleaved within an internal Tyr-encoding UAU or UAC codon, which is subsequently converted by poly(A) synthesis to the UAA termination codon, resulting in a C-terminally truncated protein variant (Yao et al. 2012). However, no eukaryotic nuclear gene that would lack a functional termination codon specified directly by the gene sequence has been found so far.

*Blastocystis* is a genus of anaerobic unicellular protists living in the intestine of various metazoans, including humans, as parasites or commensals (Clark et al. 2013). Indeed, it is probably the most prevalent protistan parasite in the human gut (Scanlan and Stensvold 2013). Its phylogenetic position had long been an enigma, but phylogenetic analyses of the 18S rRNA gene sequence (Silberman et al. 1996) and subsequent phylogenomic analyses of multigene matrices (e.g., Hampl et al. 2009) unambiguously demonstrated that it forms a lineage within a major eukaryotic assemblage called the Stramenopiles. *Blastocystis* cells have a number of unorthodox characteristics, the most notable of which are their mitochondrion-related organelles (MROs). Functionally, these organelles seem to lie halfway on a spectrum between canonical mitochondria of aerobic eukaryotes and anaerobic MROs called hydrogenosomes (Stechmann et al. 2008; Denoeud et al. 2011).

The unusual biological features of *Blastocystis* and its medical significance led to the initiation of a genome sequencing project, which was brought to fruition by Denoeud et al. (2011). Their draft genome sequence of *Blastocystis* sp. subtype 7 comprised around 18.8 Mb and harbored approximately 6,000 predicted protein-coding genes with short introns and intergenic regions. In this report, we describe a new striking aspect of *Blastocystis* molecular biology that we discovered while analyzing this genome.

## Results and Discussion

**Fig. 1.—:**
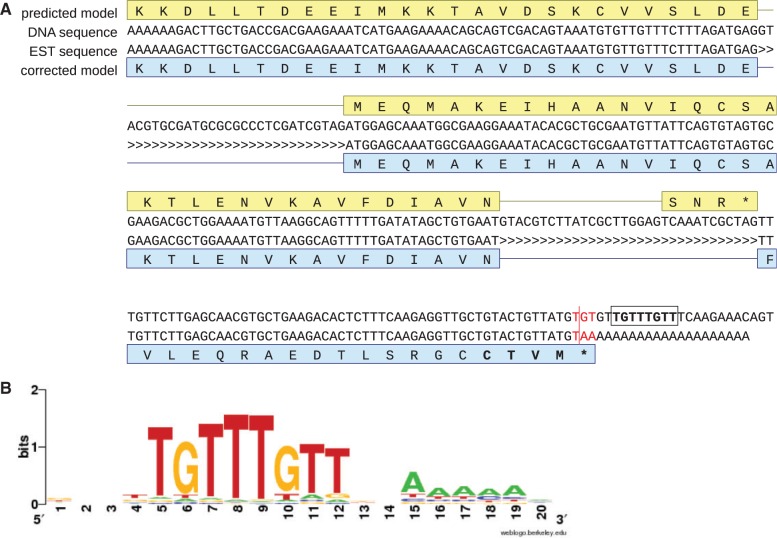
Polyadenylation-mediated creation of termination codons in *Blastocystis* sp. subtype 7. (*A*) An example of a gene from *Blastocystis* sp. subtype 7 with polyadenylation-dependent creation of a termination codon. The figure shows the 3′-end of a gene (FN668683.1, positions 539586–539266) encoding a GTPase of the RHO family, which is characterized by the presence of a C-terminal CXXX motif. Although the current prediction of the respective protein sequence (CBK24281.2) lacks the expected C-terminal motif, the protein sequence predicted by taking into account an EST sequence (FQ805196.1) does exhibit the motif (CTVM in bold). The termination codon in the EST sequence (TAA) created by polyadenylation is highlighted in red, the conserved polyadenylation motif (see the text) is boxed, introns are marked by the characters “>.” The sequences displayed in the figure are defined above by their GenBank accession numbers. (*B*) A sequence logo for the conserved motif downstream from the polyadenylation site. The logo was created using WebLogo (Crooks et al. 2004; http://weblogo.berkeley.edu/, last accessed July 15, 2014) on the basis of 2,419 individual sequences.

We were initially interested in genes of the Ras superfamily of GTPases in the genome of *Blastocystis* sp. subtype 7. Many members of this superfamily undergo functionally essential posttranslational prenylation of one or two cysteine residues close to the C-terminus (Pechlivanis and Kuhlmann 2006). However, we noticed that some of the predicted *Blastocystis* Ras superfamily GTPases lack the expected C-terminal Cys-containing motifs (fig. 1*A* and supplementary fig. S1*A*, Supplementary Material online). By inspecting the expressed sequence tag (EST) sequences for these genes, we found that termination codons were created at the transcript level by the addition of poly(A) tails downstream of particular nucleotides in the primary transcripts. Once these termination codons are considered, the predicted proteins bear prenylation motifs typical for the corresponding group of GTPases (fig. 1*A* and supplementary fig. S1*A*, Supplementary Material online).

These observations prompted us to further investigate the extent of this phenomenon in this genome. Using available EST sequences from *Blastocystis* sp. subtype 7, we were able to define the polyadenylation site for 2,419 nuclear genes (additional 226 cases of potential polyadenylation were considered as spurious by a variety of criteria—see supplementary methods, Supplementary Material online). Of these genes, 372 (i.e., ∼15%) unambiguously exhibited termination codons created by adding the poly(A) tail at a position where the underlying gene sequence did not specify a termination codon; in virtually all these genes, there was no evidence for an alternative form of the 3′-end of the transcript, suggesting that the introduction of the termination codons by polydenylation does not result from processing of aberrant or incomplete transcripts (e.g., by cytoplasmic adenylation involved in mRNA decay). Of the termination codons created this way, 359 were UAA and 13 were UGA. In addition, we identified 90 additional cases where the gene sequence specified potentially functional UAG or UGA termination codons, which were transformed into the UAA codon by polyadenylation (supplementary fig. S1*B*, Supplementary Material online).

Although the 372 genes with termination codons created by polyadenylation represent approximately 15% of all 2,419 nuclear genes with clearly defined polyadenylation sites, the number of poly(A)-containing ESTs associated with the former gene category was only 687, that is, approximately 4%, out of 16,006 poly(A)-containing ESTs mapping to the 2,419 nuclear genes. It is therefore possible that the genes with termination codons created by polyadenylation on average have a lower expression level than genes with termination codons specified directly by the gene sequence. We additionally tested the possibility that polyadenylation-mediated creation of termination codons occurs preferentially in certain functional gene categories. Using the Gene ontology (GO) framework (Ashburner et al. 2000), we found that the set of genes that use polyadenylation to create termination codons is significantly enriched (*P* value 2.0E-6, Fisher’s exact test) in genes annotated as GO:0003824—catalytic activity (i.e., genes encoding enzymes). The biological significance of this observation is unclear and analyses of all genes in the *Blastocystis* genome will be needed to confirm this bias.

Based on the foregoing estimate of approximately 15%, we assume that approximately 900 of the 6,000 annotated genes in this *Blastocystis* subtype rely on polyadenylation-mediated creation of termination codons. As this unusual phenomenon was not noticed by the annotation team of the *Blastocystis* sp. subtype 7 genome (Denoeud et al. 2011), the C-terminal regions of a large fraction of genes are predicted incorrectly; these erroneous gene models usually have either an artificial extension of the terminal exon to the first stop codon in-frame with the coding sequence (supplementary fig. S1*A*, Supplementary Material online) or an artificial intron accompanied by an artificial terminal exon that includes a hypothetical termination codon (e.g., see fig. 1*A*).

**Fig. 2.—:**
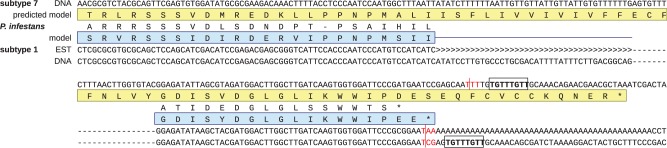
Example of a gene from *Blastocystis* sp. subtype 1 displaying polyadenylation-mediated creation of the termination codon. The termination codon in the transcript (TAA) is highlighted in red, the putative polyadenylation motif is boxed, an intron is marked by the characters “>.” Homologous proteins from *Blastocystis* sp. subtype 7 and *Phytophthora infestans* are shown for comparison. Note that the protein sequence from *Blastocystis* sp. subtype 7 is most likely incorrectly predicted, because the gene also seems to rely on polyadenylation-mediated creation of a termination codon, as is indicated by the presence of a putative polyadenylation motif (boxed). Hence, the triplet TTT (in red) is probably changed to TAA in a corresponding transcript, which would shorten the predicted coding sequence of the gene and make the encoded protein more similar to its homologs. Subtype 7 DNA sequence: FN668683.1, positions 458734–458973; subtype 7 predicted model: CBK24259.2; *Phytophthora infestans* protein sequence: XP_002895556.1; subtype 1 DNA sequence: a part of a scaffold from a genome assembly based on 454 and Illumina reads (Gentekaki E, Curtis B, Roger AJ, unpublished data); subtype 1 EST sequence: EC650050.1; subtype 1 predicted protein sequence: a theoretical inference based on the EST sequence.

We then looked for possible signals that may govern the positioning of the polyadenylation sites in *Blastocystis*. Studies of the mRNA 3′-end processing in metazoan cells established the existence of a highly conserved AAUAAA motif located 20–30 nucleotides upstream of the polyadenylation site and a less well-conserved GU-rich sequence (also called downstream sequence element, DSE) (Proudfoot 2011). Although we could not find any such conserved motif upstream of the polyadenylation sites in *Blastocystis*, we identified the conserved motif TGTTTGTT (UGUUUGUU at the RNA level), or close variants (fig. 1*B*), located four (nonconserved) nucleotides downstream of the polyadenylation site (fig. 1*A* and supplementary fig. S1, Supplementary Material online). This sequence pattern was highly conserved: Of the 2,419 nuclear genes with clearly defined polyadenylation sites, 2,333 (i.e., ∼96%) exhibited the motif located exactly four nucleotides downstream the polyadenylation site and differing from the TGTTTGTT consensus by up to two substitutions, whereas the remaining approximately 4% of cases displayed three or four substitutions in the motif and/or the motif was located three of five (instead of four) nucleotides downstream of the polyadenylation site. The conservation of the motif and its invariant distance from the polyadenylation site enabled us to revise the predictions of the 3′-end of some *Blastocystis* genes currently lacking direct transcriptomic evidence (examples are shown in fig. 2 and supplementary fig. S1*C* and *D*, Supplementary Material online).

We hypothesize that the TGTTTGTT motif functions similarly to DSE in mammals, which recruits a multisubunit complex, the cleavage stimulation factor (CstF), through the binding of its subunit CSTF2 (also called CstF-64) (Pérez Cañadillas and Varani 2003). To test this notion we looked for a possible ortholog of the human CSTF2 in *Blastocystis* and found a protein, CBK24645.2, that is the reciprocal best BLAST hit to CSTF2. However, the *Blastocystis* protein is much shorter (142 amino acid residues) than CSTF2 (577 amino acid residues) and corresponds only to its N-terminal region comprising the RNA-binding domain and the so-called hinge domain (Hockert et al. 2010). No EST evidence is available to confirm that the gene model underlying the putative CSTF2 ortholog in *Blastocystis* sp. subtype 7 is correct, but investigation of the genomic sequence downstream of the gene did not reveal a region potentially encoding the missing C-terminal part of the protein. Our unpublished transcriptomic data from *Blastocystis* sp. subtype 1 indicate that CSTF2 is indeed a short protein confined to the two N-terminal domains in the *Blastocystis* genus (data not shown). The presence of the RNA-binding domain suggests that this protein may potentially be the binding partner of the TGTTTGTT motif, but the lack of the C-terminal region including a functionally important C-terminal domain conserved at least in opisthokonts and plants (Qu et al. 2007) indicates that there may be important differences in mechanistic details of the CstF functioning between *Blastocystis* and other eukaryotes. A broader comparative analysis and direct experimental characterization of the mRNA 3′-end processing machinery in *Blastocystis* are needed to allow more specific conclusions.

As *Blastocystis* is a genetically diverse genus (Clark et al. 2013), it is of interest to know whether polyadenylation-mediated termination codon creation in nuclear mRNA transcripts is specific to subtype 7 or whether it is more widespread. To address this, we used previously published EST data (Stechmann et al. 2008) to investigate our preliminary draft genome sequence of *Blastocystis* sp. subtype 1 for the presence of genes that require this mechanism. Indeed, we found a number of cases of poly(A)-mediated termination codon creation, an example of which is portrayed in figure 2. In this case, a triplet TCG in the sequence of a gene encoding a homolog of the RPC8 subunit of the RNA polymerase III is changed in a corresponding transcript to an in-frame termination codon TAA. Furthermore, the position of the polyadenylation site in *Blastocystis* sp. subtype 1 is apparently governed by the same conserved polyadenylation motif as in the subtype 7 (fig. 2). Therefore, it is likely that poly(A)-mediated generation of termination codons is a feature that evolved either prior to, or early within, the diversification of the major subtypes of the *Blastocystis* genus. It will be interesting to investigate whether a similar mechanism occurs in related anaerobic stramenopile groups, such as opalinids and proteromonads (Silberman et al. 1996; Kostka et al. 2007).

## Conclusions

Termination codons are essential signals in mRNA molecules and their absence triggers a specific mechanism of mRNA surveillance called nonstop decay (Klauer and van Hoof 2012). It appears that in the *Blastocystis* lineage the specification of the polyadenylation site has evolved to exhibit at least three unusual properties: 1) It does not utilize a conserved upstream element; 2) it relies on a highly conserved DSE, whereas other eukaryotes in which the mRNA 3′-end processing mechanism has been investigated in sufficient detail exhibit a much higher level of degeneracy of DSE (Wahle and Rüegsegger 1999; Proudfoot 2011); and 3) it is so precise (i.e., four nucleotides upstream of the highly conserved DSE) that over time the 3′-UTRs in many genes were reduced until all that was left was the first one or two nucleotides of the termination codon. Comprehensive genomic and transcriptomic investigations of diverse *Blastocystis* lineages are needed to better understand the evolutionary origin of this phenomenon, and biochemical studies are required to elucidate the mechanistic details of the 3′-end processing of primary transcripts in *Blastocystis*.

## Materials and Methods

Briefly, the analysis used the *Blastocystis* sp. subtype 7 genome assembly version 2.0 (https://www.genoscope.cns.fr/externe/Download/Projets/Blastocystis/assembly/, last accessed July 15, 2014) and EST sequences (34,470 in total) submitted to GenBank (http://www.ncbi.nlm.nih.gov, last accessed July 15, 2014) by Genoscope. The program STAR (Dobin et al. 2013) was used to align the EST sequences to the genome sequence. In-house programs written in the Java language were then used to define polyadenylation sites and the cases where polyadenylation introduces a termination codon into the transcript. Genes exhibiting polyadenylation-mediated creation of termination codons in the *Blastocystis* sp. subtype 1 were identified in a draft genome assembly obtained from 454 and Illumina reads (Gentekaki E, Curtis B, Roger AJ, unpublished data) using EST data available for this isolate (Stechmann et al. 2008). The GO term enrichment analysis was performed using the Blast2GO tool (Conesa et al. 2005). Conserved motifs that may constitute the polyadenylation signal in a 50-bp region upstream and downstream of the mapped polyadenylation sites were searched using the qPMS7 algorithm for de novo motif search (Dinh et al. 2012; http://motifsearch.com/, last accessed July 15, 2014). Details on the analyses are provided in supplementary methods, Supplementary Material online.

## Supplementary Material

Supplementary methods and figure S1 are available at *Genome Biology and Evolution* online (http://www.gbe.oxfordjournals.org/).

Supplementary Data
