# Supplementary material for: A Large Number of Nuclear Genes in the Human Parasite Blastocystis Require mRNA Polyadenylation to Create Functional Termination Codons
Source: Genome Biol Evol. 2014 Jul 10;6(8):1956–61. doi: 10.1093/gbe/evu146 (PMC4159000; doi:10.1093/gbe/evu146)
Supplement: Supplementary Data [file supp_6_8_1956__index.html]

A Large Number of Nuclear Genes in the Human Parasite Blastocystis Require mRNA Polyadenylation to Create Functional Termination Codons — Supplementary Data 

# A Large Number of Nuclear Genes in the Human Parasite *Blastocystis* Require mRNA Polyadenylation to Create Functional Termination Codons

## Supplementary Data

files

**Files in this Data Supplement:**

- Supplementary Data - pdf file
