# Supplementary material for: A Large Number of Nuclear Genes in the Human Parasite Blastocystis Require mRNA Polyadenylation to Create Functional Termination Codons
Source: Genome Biol Evol. 2014 Jul 10;6(8):1956–61. doi: 10.1093/gbe/evu146 (PMC4159000; doi:10.1093/gbe/evu146)
Supplement: Supplementary Data [file supp_evu146_Klimes_al_SupplementalMaterial_GBE_revised.pdf]

Supplementary material for the Letter:

A large number of nuclear genes in the human parasite *Blastocystis* require mRNA polyadenylation to create functional termination codons

Vladimír Klimeš[1], Eleni Gentekaki[2], Andrew J. Roger[2,3], Marek Eliáš[1*]

[1]University of Ostrava, Faculty of Science, Department of Biology and Ecology, 710 00 Ostrava, Czech Republic. [2]Department of Biochemistry and Molecular Biology, Dalhousie University, Halifax, Nova Scotia, Canada B3H 4R2. [3]Canadian Institute for Advanced Research, Integrated Microbial Biodiversity Program.
*E-mail: marek.elias@osu.cz

Supplementary methods

Mapping ESTs onto the *Blastocystis* genome

The analysis was based on the *Blastocystis* sp. subtype 7 genome assembly version 2.0 (https://www.genoscope.cns.fr/externe/Download/Projets/Blastocystis/assembly/) and expressed sequence tag (EST) sequences (34,470 in total) submitted to GenBank by Genoscope (http://www.ncbi.nlm.nih.gov/, accession numbers FQ793876-FQ828345). We used the program STAR (Dobin et al. 2013) to align the EST sequences to the genome sequence using default parameter settings (the maximum intron size set to 2000 and the minimal chimeric segment size set to 40). The program reports the best alignment for each EST sequence. When there are more alignments with the same score for a given EST sequence, they are all reported in the output. As a result we obtained 38,511 EST-genome alignments, i.e. more than the number of ESTs entering the analysis.

2,445 alignments were discarded because of unknown bases (N) in the respective EST sequences, which would complicate further analyses. 16,243 alignments contained a potential poly(A) tail, i.e. a region of the EST not mapping to the genome (scaffold) and starting with AAAAA (or ending with TTTTT, assuming that the EST sequence is in the reverse orientation). 12 of these alignments contained a potential poly(A) tail on both ends. In total we found 16,255 potential pieces of evidence for individual polyadenylation events in *Blastocystis*.

Analysis of polyadenylation sites in *Blastocystis*

For each of the putative poly(A) tails we tried to precisely pinpoint the position of the polyadenylation site, i.e. the nucleotide immediately upstream of the poly(A) tail itself. While this site was obvious in most cases, other cases were ambiguous when the first one or several adenosines of the putative poly(A) tail aligned with adenosines in the genomic sequence. Strikingly, inspection of the sequences around the unambiguous polyadenylation sites revealed the existence of a highly conserved motif downstream of the polyadenylation site separated from it by exactly four non-conserved nucleotides (see the main text and Fig. 1B). The ambiguous cases were thus tentatively resolved by looking for a possible polyadenylation site followed by the sequence xxxxTGTTTGTT or minimally differing variants thereof.
We next looked for polyadenylation sites that probably correspond to the same gene. We clustered the sites such that the distance between neighbouring sites in each cluster was not higher than 5 nucleotides, allowing for possible sequencing errors or alternative polyadenylation sites employed by a single gene. Using this rule we got 2,645 clusters. We classified the clusters based on the number of putative different polyadenylation sites and on the presence/absence of the polyadenylation motif, which was defined as the sequence pattern TGTTTGTT or variants thereof differing by maximally two

substitutions, located downstream of the polyadenylation site separated from it by four nucleotides. The 2,645 clusters could thus be divided into the following four categories:

I. 2,135 clusters with one polyadenylation site and with the polyadenylation motif

II. 198 clusters with more than one polyadenylation site and with the polyadenylation motif associated with at least one site

III. 304 clusters with one polyadenylation site but without a typical polyadenylation motif at the expected place four nucleotides downstream of the polyadenylation site

IV. 8 clusters with more than one polyadenylation site and without a typical polyadenylation motif at the expected place

Whereas interpretation of the clusters of the first category is straightforward, the remaining three categories are more complex. We analyzed them manually to distinguish likely candidates for functional polyadenylation events from possible errors or various artifacts. The results of the manual analysis are described below.

In the 198 clusters with more than one polyadenylation site and at least one polyadenylation motif (category II), we identified the following subcategories:

IIA. 6 clusters with two polyadenylation sites each associated with the polyadenylation motif, which can be interpreted as evidence for functional alternative polyadenylation sites (for an example see Figure S1E)

IIB. 148 clusters in which a majority of ESTs indicated the same polyadenylation site four nucleotides upstream of the polyadenylation motif and a minority of ESTs (often only one) indicated a "wrongly" positioned polyadenylation site

IIC. 29 clusters with a majority of ESTs indicating a "wrongly" positioned polyadenylation site and a minority of ESTs indicating a polyadenylation site with the expected positioning four nucleotides upstream of the polyadenylation motif

IID. 15 clusters each with only two ESTs, one with a polyadenylation site four nucleotides upstream of the polyadenylation motif and one with a "wrongly" positioned polyadenylation site

In most of the cases the "wrongly" positioned polyadenylation sites were located either 3 or 5 non-conserved nucleotides upstream of the polyadenylation motif. This can reflect either an inherent inaccuracy of the polyadenylation machinery or sequencing errors; more comprehensive transcriptomic data should aid in determining the relative contribution of these different factors. Nevertheless, for all these clusters there was evidence that a polyadenylation site at the expected distance of four nucleotides upstream of the polyadenylation motif is utilized at least to some extent.

In the 304 clusters with one polyadenylation site but without a typical polyadenylation motif (category III) we identified:

IIIA. 50 clusters containing a less conserved version of the polyadenylation motif, with 3 or 4 substitutions as compared to the canonical TGTTTGTT pattern. Most of the substitutions were at the 3'-end of the motif that seems to be generally less conserved (Figure 1B in the main text).

IIIB. 31 clusters containing a typical yet "wrongly" positioned polyadenylation site located either 3 or 5 non-conserved nucleotides downstream of the polyadenylation site

IIIC. 4 clusters containing a polyadenylation site further downstream (five or more nucleotides) from the motif TGTTTGTT (or a variant thereof). Each cluster contained only one EST sequence

IIID. 58 clusters containing a polyadenylation site at a "wrong" end of the transcript, i.e. at the 3'-end of a putative transcript that is in the reverse orientation with respect to the underlying predicted gene model

IIIE. 32 clusters containing evidence for a polyadenylation site in a region of the genome, where no gene model was predicted by previous annotators (Denoeud et al. 2011) and where we could create no suitable gene model

IIIF. 88 clusters each corresponding to a gene for which another cluster exists indicating a more likely position of a functional polyadenylation site (i.e. a site associated with the conserved polyadenylation motif)

IIIG. 9 clusters containing a probably non-functional polyadenylation site but without transcriptomic evidence for an alternative polyadenylation site for the same gene. These sites were considered non-functional since they led to transcripts with either no proper termination codon or with a termination codon terminating the coding sequence prematurely (as evidenced by comparison to homologous genes). Each cluster contained only one EST sequence.

IIIH. 32 clusters containing a polyadenylation site lacking the conserved polyadenylation motif downstream, but without other obvious hints of being non-functional

In the 8 clusters with more than one polyadenylation site and without a typical polyadenylation motif at the expected place (category IV) we identified:

IVA. 3 clusters with a majority of ESTs showing the polyadenylation site four nucleotides upstream of a putative polyadenylation motif differing by 3 substitutions from the most frequent pattern, and with only a minority of ESTs "wrongly" positioned with respect to the motif

IVB. 1 cluster with a majority of ESTs indicating a "wrongly" positioned polyadenylation site and a minority of ESTs indicating a polyadenylation site with the expected positioning four nucleotides upstream of the polyadenylation motif, although the motif differed by 3 substitutions from the typical TGTTTGTT pattern

IVC. 1 cluster containing an example of an alternative polyadenylation with both polyadenylation sites in the cluster positioned at the expected distance from a putative polyadenylation motif, albeit the motif in both cases bears 3 substitutions as compared to the typical TGTTTGTT pattern

IVD. 1 cluster containing polyadenylation sites on the "wrong" end of the transcript (i.e. the 5'-end) based on the predicted gene model

IVE. 2 clusters each corresponding to a gene for which another cluster exists that indicates a more likely position of a functional polyadenylation site (i.e. a site associated with the conserved polyadenylation motif)

Based on the analysis above, cluster categories I, II, IIIA, IIIB, IVA, IVB, and IVC were considered as bearing evidence for functional polyadenylation events directed by the putative polyadenylation motif-based mechanism (characterized by the presence of a conserved polyadenylation motif TGTTTGTT or its variants differing by no more than four substitutions and located 3-5 nucleotides

downstream of the polyadenylation site). Altogether this gives 2,419 clusters with clearly defined polyadenylation sites. The remaining 226 clusters were considered to reflect spurious polyadenylation cases, the significance of which cannot be presently ascertained (but we suspect that most of them probably correspond to accidentally polyadenylated, incomplete, partially degraded or non-coding transcripts).

Analysis of polyadenylation-mediated creation of termination codons in *Blastocystis*

Motivated by the serendipitous observation of *Blastocystis* genes that employ transcript polyadenylation to create termination codons (see the main text), we explored the 2,419 clusters with clearly defined polyadenylation sites (see previous section) to identify these cases on a systematic basis. In the first step, we specifically looked for clusters with the following properties:

- The nucleotide at the polyadenylation site must be T, A preceded by T, or G preceded by T, because only in those cases polyadenylation leads to formation of TAA or TGA codons (the termination codon TAG cannot be formed by polyadenylation in principle).
- There is a reading frame in an EST sequence in the cluster that does not contain any termination codon up to an in-frame TAA or TGA triplet with an least one of the adenosines coming from the poly(A) tail, or the EST contains an open reading frame at least half the size of the EST length, starting with an ATG triplet and extending to an in-frame TAA or TGA triplet with an least one of the adenosines coming from the poly(A) tail.
- There is at least one EST with these properties in the cluster.

563 clusters meeting the criteria specified above were analyzed manually to distinguish positive cases of polyadenylation-mediated creation of termination codons from possible false positive cases. For clusters overlapping with an existing protein-coding gene model we checked whether the potential termination codon created by polyadenylation is indeed in-frame with the predicted coding sequence of the gene or whether it was in another reading frame. If there was no gene model predicted in the respective region of the genome, we tried to deduce a probable novel gene model from the genomic sequence and then checked whether the potential termination codon created by polyadenylation is in-frame with the predicted coding sequence. We thus identified:

- 372 sites where polyadenylation created the actual termination codon of the mature transcript
- 1 site that formed a possible alternative termination codon in the middle of the predicted model, which would result in an alternative shorter version of the encoded protein
- 9 sites associated with two possible overlapping gene models in different reading frames where it was not possible to decide with certainty which of the reading frames is the genuine one (and hence the termination codon created by polyadenylation is actually used as such)
- 92 sites, where polyadenylation changed a potentially functional termination codon specified by the gene sequence to another termination codon (e.g. TGA to T|AA, where the vertical line indicates the boundary between the original transcript and the poly(A) tail)
- 35 sites, where polyadenylation most likely replaced one or both adenosines of a termination codon originally present in the primary transcript (so that the termination codon in the mature polyadenylated transcript remained the same); these events were inferred considering the presumed characteristic distance between the polyadenylation site and the conserved polyadenylation motif

- 50 sites, where polyadenylation created TAA or TGA triplets, but apparently in a wrong (i.e. non-translated) reading frame, so these triplets are unlikely to function as actual termination codons

- 1 site that was located in a cluster with multiple polyadenylation sites and where a different site was considered to be the “right” one (i.e. the one associated with the conserved polyadenylation motif)

- 3 sites that predicted a false termination codon because of a frame-shift error in the EST reads

Altogether, we identified at least 372 (but potentially up to 382 if the ambiguous second and third categories prove to be genuine) genes that employ transcript 3'-end processing and polyadenylation to create functional termination codons.

GO term enrichment analysis
For each of the 2,645 clusters of polyadenylation sites we took the longest EST sequence, yielding 2,551 different EST sequences (the difference in the numbers is because some ESTs had poly(A) tails on both ends and because some ESTs align to multiple places in the genome a thus belong to more than one cluster). These ESTs were used as queries for blastx searches (Altschul et al. 1997) and the outputs were analysed using the Blast2GO tool (Conesa et al. 2005) to assign GO terms to the sequences. 1,429 EST sequences were annotated with GO terms, of which 207 represented genes with termination codons formed by polyadenylation. We used the Gossip package (Blüthgen et al. 2005) integrated in Blast2GO to statistically asses the differences between the annotation of the 207 sequences and the remaining sequences. This package uses the Fisher’s Exact Test and corrects for multiple testing. This analysis revealed that the two sequence categories differ only in the GO term 0003824 (catalytic activity), which was significantly enriched in the set of genes with polyadenylation-mediated formation of termination codons (62% versus 44% genes annotated with this GO term).

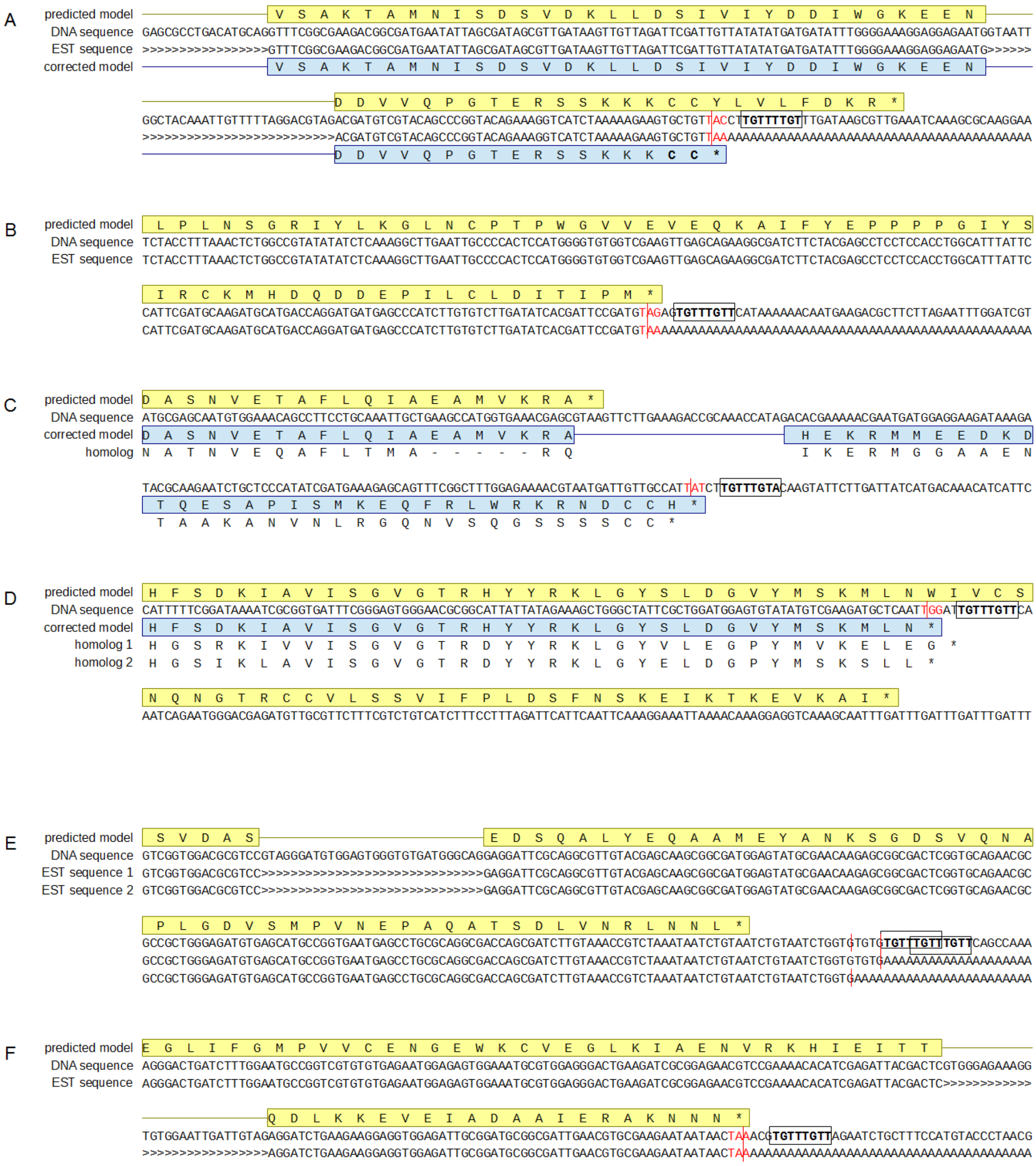

Figure S1. Examples of genes from *Blastocystis* sp. subtype 7 with polyadenylation-mediated creation of termination codons. Sequence identifiers indicated below all represent GenBank accession numbers.

(A) A gene encoding an ortholog of Rab32, a GTPase of the RAB family. RABs are characterized by the presence of two (rarely one) cysteine residues near the very C-terminus of the proteins, most often in the configurations CC*, CXC*, CCX*, or CCXX* (where * indicates the free C-terminus of the protein; Pechlivanis and Kuhlmann 2006). The two Cys residues are geranylgeranylated through the action of a RAB-specific geranylgeranyl transferase II (GGTase II) and this post-translational modification is essential for the proper function of RAB proteins (Pechlivanis and Kuhlmann 2006). The predicted protein sequence of Rab32 from *Blastocystis* sp. subtype 7 (CBK21164.2) has an unusually high number of amino acid residues downstream of the two Cys residues, raising doubts

about the effectiveness of the GGTase II toward this substrate. However, when the gene model is revised by considering the EST sequence (FQ825458.1) covering the 3'-end of the gene, the resulting protein sequence prediction exhibits a standard double-Cys motif at the C-terminus. Note that the termination codon in the EST sequence (TAA in red) is created by polyadenylation, since the corresponding DNA sequence is TAC. The position of the polyadenylation site is governed by the conserved polyadenylation motif (boxed letters in bold); the vertical line indicates the boundary between the region of the primary transcript kept in the mature transcript and the polyA tail. DNA sequence: FN668640.1, positions 493498-493737 (reverse complement).

(B) An example of a gene for which polyadenylation changes the type of the termination codon. In this case the potentially functional termination codon TAG specified by the genome sequence is changed to the TAA codon by cleaving the primary transcript immediately downstream of the T nucleotide and adding the poly(A) tail. DNA sequence: FN668647.1, positions 59370-59609 (reverse complement); EST sequence: FQ824200.1; predicted model: CBK21164.2.

(C) An example of a gene currently lacking transcriptomic evidence, the structure of which could be refined by taking into account the conserved polyadenylation motif. The gene codes for a RAB family GTPase representing the Rab1A paralog specific for the SAR clade (Elias et al. 2009). As other RABs, the encoded protein is expected to possess a C-terminal Cys-containing prenylation motif, but the protein sequence currently predicted for the gene lacks it. Addition of an extra exon to the gene model, in which the putative polyadenylation motif (boxed) would govern a polyadenylation-mediated change of the triplet TAT to a termination codon TAA, revises the sequence of the predicted protein so that it now includes the typical C-terminal extension with two Cys residues close to the very C-terminus. A homologous RAB protein sequence (from *Yarrowia lipolytica*) is included in the alignment to show a better fit of the revised protein prediction to the canonical structure of a RAB GTPase. DNA sequence: FN668647.1, positions 114950-115189 (reverse complement); predicted model: CBK22118.2; homolog: XP_502561.1.

(D) An example of a gene currently lacking transcriptomic evidence, the structure of which could be refined by taking into account the conserved polyadenylation motif. The gene codes for a homolog of the elongator complex protein 3 (ELP3 family of histone acetyltransferases). The current prediction of the gene in *Blastocystis* sp. subtype 7 includes an unusual C-terminal extension beyond the highly conserved C-terminus of homologs from other species (only two are shown for simplicity). When a putative polyadenylation site (red vertical line) predicted based on the location of the putative polyadenylation motif (boxed) is considered, a new termination codon is created which makes the protein sequence shorter and fitting in length to the rest of the homologs. DNA sequence: FN668638.1, position 1462896-1463135 (reverse complement); predicted model: CBK20094.2; homolog 1: XP_004346367.1 (*Capsaspora owczarzaki* ATCC 30864); homolog 2: EQC38571.1 (*Saprolegnia diclina* VS20).

(E) An example of a gene with evidence for utilizing alternative polyadenylation sites. Note that both polyadenylation sites are followed by the putative polyadenylation motif. DNA sequence: FN668647.1, positions 53810-54049 (reverse complement); EST sequence 1: FQ827052.1, the same polyadenylation site is also supported by ESTs FQ799545.1 and FQ799494.1; EST sequence 2: FQ827756.1, the same polyadenylation site is also supported by ESTs FQ801532.1, FQ798686.1 and FQ798004.1; predicted model: CBK22087.2.

(F) An example of a gene for which polyadenylation putatively replaces the last adenosine residue in the termination codon, so that the termination codon remains the same as in the primary transcript. DNA sequence: FN668639.1, positions 730650-730889; EST sequence: FQ805656.1; predicted model: CBK20544.2.

Supplementary references

Altschul SF, Madden TL, Schäffer AA, Zhang J, Zhang Z, Miller W, Lipman DJ. 1997. Gapped BLAST and PSI-BLAST: a new generation of protein database search programs. Nucleic Acids Res 25:3389-3402.

Blüthgen N, Brand K, Cajavec B, Swat M, Herzel H, Beule D. 2005. Biological profiling of gene groups utilizing gene ontology. Genome Inform 16:106-115.

Conesa A, Götz S, Garcia-Gomez JM, Terol J, Talon M, Robles M. 2005. Blast2GO: a universal tool for annotation, visualization and analysis in functional genomics research. Bioinformatics 21:3674-3676.

Denoeud F, Roussel M, Noel B, Wawrzyniak I, Da Silva C, Diogon M, Viscogliosi E, Brochier-Armanet C, Couloux A, Poulain J, Segurens B, Anthouard V, Texier C, Blot N, Poirier P, Ng GC, Tan KS, Artiguenave F, Jaillon O, Aury JM, Delbac F, Wincker P, Vivarès CP, El Alaoui H. 2011. Genome sequence of the stramenopile *Blastocystis*, a human anaerobic parasite. Genome Biol 12:R29.

Dobin A, Davis CA, Schlesinger F, Drenkow J, Zaleski C, Jha S, Batut P, Chaisson M, Gingeras TR. 2013. STAR: ultrafast universal RNA-seq aligner. Bioinformatics 29:15-21.

Elias M, Patron NJ, Keeling PJ. 2009. The RAB family GTPase Rab1A from *Plasmodium falciparum* defines a unique paralog shared by chromalveolates and rhizaria. J Eukaryot Microbiol 56:348-356.

Pechlivanis M, Kuhlmann J. 2006. Hydrophobic modifications of Ras proteins by isoprenoid groups and fatty acids--More than just membrane anchoring. Biochim Biophys Acta 1764:1914-1931.
